# Government trust, perceptions of COVID-19 and behaviour change: cohort surveys, Singapore

**DOI:** 10.2471/BLT.20.269142

**Published:** 2020-10-28

**Authors:** Vanessa W Lim, Rachel L Lim, Yi Roe Tan, Alexius SE Soh, Mei Xuan Tan, Norhudah Bte Othman, Sue Borame Dickens, Tun-Linn Thein, May O Lwin, Rick Twee-Hee Ong, Yee-Sin Leo, Vernon J Lee, Mark IC Chen

**Affiliations:** a Infectious Disease Research and Training Office, National Centre for Infectious Diseases, 16 Jln Tan Tock Seng, Singapore 308442, Singapore.; b Saw Swee Hock School of Public Health, National University of Singapore, Singapore, Singapore.; c Wee Kim Wee School of Communication and Information, Nanyang Technological University, Singapore, Singapore.; d Communicable Diseases Division, Ministry of Health, Singapore, Singapore.

## Abstract

**Objective:**

To evaluate how public perceptions and trust in government communications affected the adoption of protective behaviour in Singapore during the coronavirus disease 2019 (COVID-19) pandemic.

**Methods:**

We launched our community-based cohort to assess public perceptions of infectious disease outbreaks in mid-2019. After the first case of COVID-19 was reported in Singapore on 23 January, we launched a series of seven COVID-19 surveys to both existing and regularly enrolled new participants every 2 weeks. As well as sociodemographic properties of the participants, we recorded changing responses to judge awareness of the situation, trust in various information sources and perceived risk. We used multivariable logistic regression models to evaluate associations with perceptions of risk and self-reported adopted frequencies of protective behaviour.

**Findings:**

Our cohort of 633 participants provided 2857 unique responses during the seven COVID-19 surveys. Most agreed or strongly agreed that information from official government sources (99.1%; 528/533) and Singapore-based news agencies (97.9%; 522/533) was trustworthy. Trust in government communication was significantly associated with higher perceived threat (odds ratio, OR: 2.2; 95% confidence interval, CI: 1.6–3.0), but inversely associated with perceived risk of infection (OR: 0.6; 95% CI: 0.4–0.8) or risk of death if infected (OR: 0.6; 95% CI: 0.4–0.9). Trust in government communication was also associated with a greater likelihood of adopting protective behaviour.

**Conclusion:**

Our findings show that trust is a vital commodity when managing an evolving outbreak. Our repeated surveys provided real-time feedback, allowing an improved understanding of the interplay between perceptions, trust and behaviour.

## Résumé

**Objectif:**

Évaluer dans quelle mesure la perception du public et la confiance envers les communications du gouvernement ont influencé l'adoption d'un comportement de protection à Singapour durant la pandémie de maladie à coronavirus (COVID-19).

**Méthodes:**

Nous avons lancé notre cohorte communautaire afin d'identifier la manière dont le public percevait les épidémies de maladies infectieuses au milieu de l'année 2019. Après l'annonce du premier cas de COVID-19 à Singapour le 23 janvier, nous avons débuté une série de sept sondages sur la COVID-19 auprès de participants existants et de nouveaux inscrits à intervalles réguliers, toutes les deux semaines. Nous avons pris note des caractéristiques sociodémographiques de ces participants ainsi que de l'évolution des réponses afin de déterminer le degré de sensibilisation à la situation, la confiance vis-à-vis de diverses sources d'information et le risque perçu. Nous avons employé des modèles de régression logistique multivariée pour examiner les liens qui existent entre la perception du risque et la fréquence à laquelle les répondants déclarent avoir adopté un comportement de protection.

**Résultats:**

Notre cohorte de 633 participants a fourni 2857 réponses uniques lors des sept sondages sur la COVID-19. La plupart étaient d'accord ou totalement d'accord pour dire que les informations provenant de sources gouvernementales officielles (99,1%; 528/533) et d'agences de presse établies à Singapour (97,9%; 522/533) étaient fiables. La confiance envers la communication du gouvernement était étroitement liée à l'intensité de la menace ressentie (odds ratio, OR: 2,2; intervalle de confiance de 95%, IC: 1,6–3,0), mais inversement proportionnelle au risque d'infection perçu (OR: 0,6; IC de 95%: 0,4–0,8) ou au risque de décès en cas d'infection (OR: 0,6; IC de 95%: 0,4-0,9). Cette confiance était également associée à une probabilité accrue d'adoption d'un comportement de protection.

**Conclusion:**

Nos résultats montrent que la confiance est un outil indispensable pour gérer la progression d'une épidémie. Les multiples études réalisées par nos soins ont livré un retour d'expérience en temps réel qui a permis de mieux comprendre les interactions entre perception, confiance et comportement.

## Resumen

**Objetivo:**

Evaluar cómo las percepciones y la confianza del público en las comunicaciones del gobierno influyeron en la adopción de un comportamiento protector en Singapur durante la pandemia de la enfermedad por coronavirus de 2019 (COVID-19).

**Métodos:**

A mediados de 2019, se inició el estudio de cohorte de la población local para evaluar la percepción que tiene el público sobre los brotes epidémicos de las enfermedades infecciosas. Tras la notificación del primer caso de la COVID-19 en Singapur el 23 de enero, se iniciaron una serie de siete sondeos sobre la COVID-19, tanto a personas que ya estaban participando como a personas nuevas que se inscribían con regularidad, cada dos semanas. Además de las propiedades sociodemográficas de las personas participantes, se registraron las nuevas respuestas para juzgar el conocimiento de la situación, la confianza en las diversas fuentes de información y la percepción del riesgo. Se emplearon modelos de regresión logística multivariante para evaluar las asociaciones con las percepciones de riesgo y las frecuencias adoptadas de comportamientos protectores que facilitaron las personas participantes.

**Resultados:**

La cohorte de esta investigación integrada por 633 personas aportó 2857 respuestas únicas durante los siete sondeos de la COVID-19. La mayoría estuvo de acuerdo o muy de acuerdo en que la información de las fuentes oficiales del gobierno (99,1 %; 528/533) y de las agencias de noticias con sede en Singapur (97,9 %; 522/533) era fiable. La confianza en las comunicaciones del gobierno se asoció de manera significativa con una mayor percepción de amenaza (oportunidad relativa, OR: 2,2; intervalo de confianza del 95 %, IC: 1,6-3,0), pero se asoció de manera inversa con la percepción de riesgo de infección (OR: 0,6; IC del 95 %: 0,4-0,8) o de riesgo de muerte si había contagio (OR: 0,6; IC del 95 %: 0,4-0,9). La confianza en las comunicaciones del gobierno también se asoció con una mayor probabilidad de adoptar comportamientos protectores.

**Conclusión:**

Los resultados indican que la confianza es un elemento vital para gestionar un brote epidémico en evolución. Los estudios que se realizaron de manera repetida aportaron información en tiempo real, lo que permitió mejorar la comprensión de la interacción entre las percepciones, la confianza y el comportamiento.

## ملخص

الغرض تقييم مدى تأثير التصورات العامة والثقة في الاتصالات الحكومية على تبني السلوك الوقائي في سنغافورة أثناء جائحة مرض فيروس كورونا (كوفيد 19).

الطريقة أطلقنا مجموعتنا المجتمعية لتقييم التصورات العامة لحالات تفشي هذا المرض المعدي في منتصف عام 2019. وبعد الإبلاغ عن الحالة الأولى للإصابة بكوفيد 19 في سنغافورة في 23 يناير/كانون ثاني، أطلقنا سلسلة من المسوح السبعة عن كوفيد 19 لكل من المشاركين الجدد الحاليين والمسجلين بانتظام كل أسبوعين. وبالإضافة إلى الخصائص الاجتماعية الديموغرافية للمشاركين، قمنا بتسجيل استجابات متغيرة للحكم على الوعي بالموقف، والثقة في مصادر المعلومات المتنوعة والمخاطر المتصورة. وقمنا باستخدام نماذج تحوّف لوجيستي متعدد المتغيرات لتقييم الارتباطات مع تصورات المخاطر وأنماط التكرار المتبعة، والتي تم الإبلاغ عنها ذاتيًا، للسلوك الوقائي.

النتائج قدمت مجموعتنا المكونة من 633 مشاركًا، 2857 استجابة فريدة أثناء المسوح السبعة حول كوفيد 19. وافقت الغالبية، أو وافقت بشكل مؤكد، على أن المعلومات الواردة من المصادر الحكومية الرسمية (99.1%؛ 528/533)، وأن وكالات الأنباء في سنغافورة (97.9%؛ 522/533) كانت جديرة بالثقة. ارتبطت الثقة في الاتصالات الحكومية بشكل كبير بالتهديد الملموس بشكل أكبر (نسبة الاحتمالات: 2.2؛ فاصل الثقة 95%: 1.6 إلى 3.0) ولكنها ارتبطت بشكل عكسي مع الخطر المتصور للإصابة بالعدوى (نسبة الاحتمالات: 0.6؛ ‏بفاصل ثقة 95%: 0.4 إلى 0.8) أو خطر الوفاة في حالة الإصابة بالعدوى (نسبة الاحتمالات: 0.6؛ ‏بفاصل ثقة 95%: 0.4–0.9). كما ارتبطت الثقة في الاتصالات الحكومية باحتمالية أكبر لاتباع السلوك الوقائي.

الاستنتاج توضح النتائج التي توصلنا إليها أن الثقة هي ضرورة حيوية عند إدارة حالة التفشي الجارية. قدمت المسوح المتكررة الخاصة بنا ملاحظات في الوقت الفعلي، مما سمح بتحقيق فهمًا أفضل للتفاعل بين التصورات والثقة والسلوك.

## 摘要

**目的:**

旨在评估在冠状病毒病（新型冠状病毒肺炎）大流行期间，公众对政府通信的看法和信任如何影响新加坡采取保护措施。

**方法:**

我们发起了基于社区的群组研究，以评估公众对 2019 年年中传染病疫情的看法。1 月 23 日新加坡报告首例新型冠状病毒肺炎病例后，我们发起了一系列的（7 项）新型冠状病毒肺炎调查，每 2 周对现有的参与者和定期招募的新参与者进行一次调查。结合参与者的社会人口统计特征，我们记录了不断变化的应对方式以判断是否了解情况、信任各种信息源和感知风险。我们采用多变量逻辑回归模型评估风险感知与自我报告保护行为实施频率之间的关联。

**结果:**

我们的 633 名参与者在 7 项新型冠状病毒肺炎调查中提供了 2857 份独特的答案。大多数人赞同或非常赞同政府提供的官方信息（99.1%；528/533）和新加坡本地新闻机构提供的信息（97.9%；522/533）值得信赖。对政府通信的信任与较高的感知威胁显著相关（比值比，OR：2.2；95% 置信区间，CI：1.6-3.0），但与感知的感染风险（OR：0.6；95% CI：0.4–0.8）或感染后的死亡风险（OR：0.6；95% CI：0.4-0.9）成反比。对政府通信的信任也与更有可能采取保护行为相关。

**结论:**

我们的研究结果表明，在处理不断发展的疫情时，信任是至关重要的因素。我们反复进行的调查提供了实时反馈，让人可以更好地理解感知、信任和行为之间的相互影响。

## Резюме

**Цель:**

Оценить, как общественное мнение и доверие к заявлениям правительства повлияли на принятие мер защитного поведения в Сингапуре во время пандемии коронавируса (COVID-19).

**Методы:**

В середине 2019 года из местного населения была сформирована когорта для оценки общественного мнения о вспышках инфекционных заболеваний. После того как 23 января в Сингапуре был зарегистрирован первый случай заболевания COVID-19, авторы запустили серию из семи опросов по COVID-19, проводимых каждые 2 недели, для существующих и регулярно набираемых новых участников. Помимо социально-демографических характеристик участников, авторы регистрировали изменения в ответах, чтобы оценить осведомленность о ситуации, доверие к различным источникам информации и предполагаемый риск. Авторы использовали многовариантные модели логистической регрессии для оценки ассоциаций с восприятием риска и принятой частотой применения мер защитного поведения, о которой сообщали участники.

**Результаты:**

Когорта из 633 участников предоставила 2857 уникальных ответов в ходе семи опросов по COVID-19. Большинство участников согласились или полностью согласились с тем, что информация из официальных государственных источников (99,1%; 528/533) и сингапурских информационных агентств (97,9%; 522/533) заслуживает доверия. Доверие к заявлениям правительства было в значительной степени связано с более высокой предполагаемой угрозой (отношение шансов, ОШ: 2,2; 95%-й доверительный интервал, ДИ: 1,6–3,0), но обратно пропорционально связано с предполагаемым риском заражения (ОШ: 0,6; 95%-й ДИ: 0,4–0,8) или с риском смерти в случае заражения (ОШ: 0,6; 95%-й ДИ: 0,4–0,9). Доверие к заявлениям правительства также было связано с большей вероятностью принятия мер защитного поведения.

**Вывод:**

Полученные результаты свидетельствуют о том, что доверие является жизненно важным ресурсом в борьбе с развивающейся вспышкой. Неоднократные опросы обеспечили обратную связь в режиме реального времени, позволяя лучше понять взаимосвязь между восприятием, доверием и поведением.

## Introduction

The emergence of coronavirus disease 2019 (COVID-19) in China in December 2019 resulted in a global and rapidly rising number of cases and deaths.^1^ Many countries responded with restrictive measures of various degrees to suppress transmission.^2^^,^^3^ However, because non-compliance and outright protests against more restrictive measures have been widespread,^4^ understanding the factors that facilitate public adherence to such interventions is important.

Singapore, a city-state in South-East Asia and a global travel hub, reported its first imported COVID-19 case in a traveller from China on 23 January 2020.^5^ Several clusters of local transmission followed, but were successfully contained without widespread use of socially disruptive measures.^6^ Singapore subsequently experienced a second wave of infections from imported cases, rising local transmission and large outbreaks in migrant worker dormitories, with the number of confirmed cases exceeding 926 by 31 March 2020.^7^ As cases continued to increase, the government announced the implementation of a so-called circuit-breaker to interrupt COVID-19 transmission on 4 April 2020. This package of measures and restrictions, combined with penalties for non-compliance,^8^^,^^9^ was equivalent to a partial lockdown. Originally set to last from 7 April to 4 May 2020, the restrictions were extended until 1 June 2020; a phased return to pre-pandemic life was instigated after this date.

An emerging global issue is how to effectively communicate and ensure the adoption of public health recommendations and containment measures.^10^ During the Ebola virus disease outbreak in Liberia in 2014, proper risk communication and health promotion encouraged community support and involvement, playing an important role in the adoption of key protective behaviour.^11^ However, studies are lacking that demonstrate the role of effective communication and trust in the perceptions of, and protective behaviour adopted in response to, COVID-19. We need tools to rapidly assess public perceptions, not just at singular time points but across multiple time points as the pandemic evolves. We describe insights from a cohort-based study to record changes in public opinions and adopted behaviour in Singapore during the COVID-19 outbreak. We examine how perceptions of the disease and the local situation, as well as trust in the government’s communications about the outbreak, affect the adoption by the general public of recommended behaviour.

## Methods

### Study design

Before the COVID-19 pandemic, we launched the community-based cohort study “Strengthening our community’s resilience against threats from emerging infections” (SOCRATEs)^12^ to periodically assess public knowledge and perceptions of common infectious diseases (e.g. dengue and tuberculosis) and previous outbreaks. Launched on 27 June 2019, our study was designed so that the cohort could be rapidly re-surveyed in the event of a new outbreak. Consenting participants aged 16 years or older were enrolled by a team of eight public health students of the National University of Singapore during their internships, trained and supervised by a research team from the National Centre for Infectious Diseases, Singapore. The research team took over the recruitment process when the internships were over. Participants were enrolled via a combination of door-to-door recruitment of the general population and self-referred participants, who received study information via word-of-mouth and social media posts. For door-to-door recruitment, we divided Singapore into five geographical zones and randomly selected an equal number of residential buildings within each zone using verified postal codes (available in data repository).^13^ We set a maximum of four participants per household. From the launch of the study until 22 January 2020, we conducted face-to-face interviews during which participants provided responses to a baseline survey and a survey about previous outbreaks of infectious diseases in Singapore. 

**Fig. 1:**
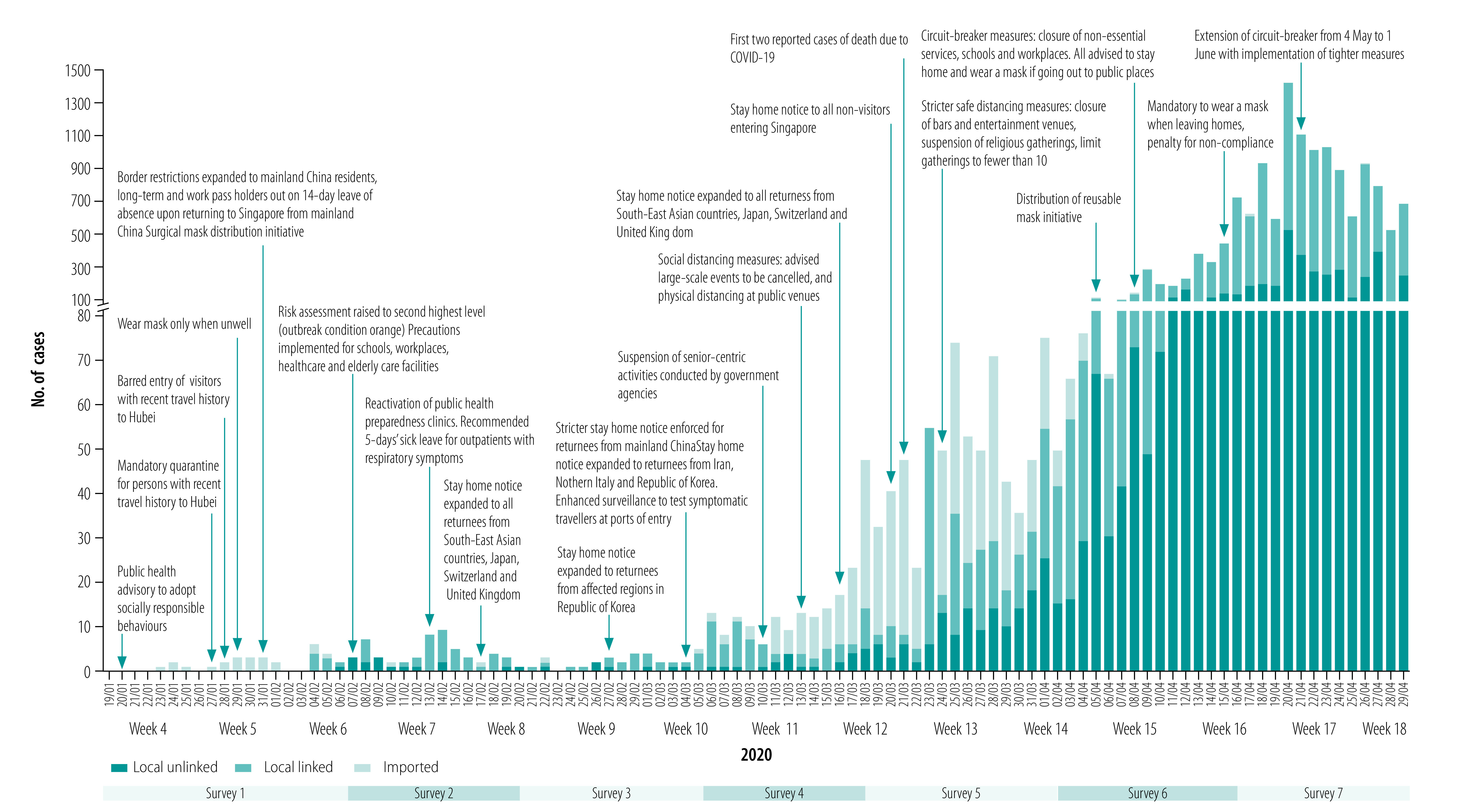
Timeline of COVID-19 pandemic, imposed measures and seven cohort surveys to assess public perceptions and behaviour, Singapore, January–April 2020

We replaced the initial outbreak survey with the first COVID-19 survey on 24 January 2020, a day after the first confirmed case of COVID-19 in Singapore (Fig. 1). From this date onwards, we conducted all surveys by messaging a link to participants’ mobile phones, and replaced door-to-door recruitment with video interviews with self-referred participants. To record changes in participants’ perceptions of, and protective behaviour adopted in response to, the pandemic, we issued our outbreak survey seven different times until 29 April 2020 (Table 1). The same basic questions (available in data repository)^13^ were included in all seven surveys, with the addition of single-use questions at certain times to (i) assess awareness of current developments and (ii) gauge responses to, and support for, government initiatives (data repository).^13^

COVID-19: coronavirus disease.

Note: The *y*-axis scale changes from 80–100 cases upwards.

**Table 1:** Start and end dates, cohort size and number of respondents to seven surveys during COVID-19 pandemic, Singapore, January–April 2020

Variable	Survey no.						
	1	2	3	4	5	6	7
Start of survey	24 Jan	6 Feb	20 Feb	5 Mar	18 Mar	2 Apr	15 Apr
End of survey	6 Feb	19 Feb	4 Mar	17 Mar	1 Apr	16 Apr	29 Apr
No. existing participants	393	437	440	440	473	520	584
No. new participants	44	3	0	33	47	64	49
Total cohort size (no. survey invites)	437	440	440	473	520	584	633
No. respondents	340	340	346	389	428	479	535

COVID-19: coronavirus disease 2019.

Recruitment continued throughout the surveys, and those who missed any particular survey could participate in the following survey. Newly recruited participants provided responses to both a baseline survey and the current COVID-19 survey. 

All surveys were available in all three key local languages (English, Mandarin and Malay).

### Data analysis

Because the start and end dates of epidemiological week numbers do not correspond to the start and end dates of surveys, we aggregated responses by epidemiological week number (where week 4 is 19–25 January 2020) based on the timestamps at submission to illustrate changes in perception and behaviour as the outbreak evolved. We calculated the proportion who selected a particular response as a percentage of the total number of responses received for that question (which varied as a result of different questions being included at different surveys, as well as a varying number of missing responses to any particular question within a particular survey), and compared these using *χ^2^* tests. We used multivariable logistic regression models to evaluate associations between various factors and perceptions of (i) the threat posed by COVID-19, (ii) the risk of infection and (iii) the risk of death upon contracting COVID-19, and between various factors and self-reported adopted frequencies of behaviour that may mitigate risk. We assessed and adjusted for various factors using a multilevel modelling framework, with a random intercept term to model the effects of participants’ behaviour. 

We considered sociodemographic properties and baseline survey responses regarding degree of trust in information from various sources to be static (level 1) variables, and other survey responses (e.g. awareness of the local situation) to be time-varying (level 2) variables. To investigate whether the stage of the epidemic influenced perceptions and self-reported behaviour, we also included variables for the number of weeks elapsed since the first case and the natural log of the numbers of new cases and cumulative deaths reported up to the day before each survey response. 

Finally, we tested the hypothesis that respondents who expressed greater trust in the government’s communications on COVID-19 would be more likely to adopt the recommended behaviour. As government recommendations on protective behaviour evolved during the outbreak, we stratified the effect over three key time periods: (i) before the Disease Outbreak Response System Condition^14^ was upgraded from yellow to orange, when messaging largely focused on personal hygiene and socially responsible behaviour to adopt if infected; (ii) after outbreak condition orange was declared on 7 February 2020, when avoidance of large-scale gatherings was recommended;^15^ and (iii) after a key speech on 4 April 2020 by the Prime Minister of Singapore regarding the circuit-breaker period. In this speech, the Prime Minister not only articulated plans for a partial lockdown, but also announced a change in guidance for facemasks (to be worn in public by all persons, superseding previous guidance that facemasks were mainly for those who were unwell).^16^^,^^17^ Results were expressed as odds ratios (ORs) with 95% confidence intervals (CIs). 

We applied multiple imputation for several exposure variables with missing responses (ranging from 0.2%; 5/2857 to 16.6%; 474/2857) based on each participant’s most recent survey response and the mean response for that question (data repository).^13^

We performed all analyses using Stata software version 15.0 (StataCorp, College Station, United States of America).

### Ethics

The Ethics Review Board of the National Healthcare Group, Singapore, approved this study (reference no. 2018/01203).

## Results

We depict the periods spanned by the seven pandemic surveys in relation to the rising number of cases and timeline of measures implemented in Singapore, as well as epidemiological week numbers, in Fig. 1. We analysed a total of 2857 survey responses from the seven surveys, in a cohort of 633 participants from 540 households (Table 2). We enrolled 33.2% (210/633) of the cohort participants via door-to-door recruitment before the COVID-19 outbreak, and 28.9% (183/633) and 37.9% (240/633) referred themselves to us before and after the COVID-19 outbreak, respectively. Our study population was evenly spread throughout the residential areas of Singapore **(**data repository).^13^ However, participants aged 45 years or older were underrepresented according to the age structure of the Singapore general population,^18^ and the number of responses to the COVID-19 surveys from the oldest age group (≥ 60 years) was lower (13.5%; 385/2857) than in the initial cohort (19.1%; 75/393). Our cohort included more women (60.8%; 385/633) than men, but ethnic distributions were comparable to those of Singapore.^18^ Most of our cohort participants were employed (71.1%; 450/633), and 12.3% (78/633) were students. 

**Table 2:** Sociodemographic characteristics of study participants in assessment of COVID-19 perceptions and corresponding behaviour, Singapore, June 2019–April 2020

Sociodemographic characteristics	No. (%)		
	Recruited during 27 June 2019–20 January 2020 (*n* = 393)^a^	Total participants over seven COVID-19 surveys (*n* = 633)^b^	Total responses over seven COVID-19 surveys (*n* = 2857)^b^
**Recruitment method**			
Door-to-door	210 (53.4)	210 (33.2)	976 (34.2)
Referral	183 (46.6)	423 (66.8)	1881 (65.8)
**Age, years**			
17–29	112 (28.5)	188 (29.7)	918 (32.1)
30–44	136 (34.6)	215 (34.0)	998 (34.9)
45–59	70 (17.8)	120 (19.0)	556 (19.5)
60–87	75 (19.1)	110 (17.4)	385 (13.5)
**Sex**			
Male	158 (40.2)	248 (39.2)	1107 (38.7)
Female	235 (59.8)	385 (60.8)	1750 (61.3)
**Ethnicity**			
Chinese	283 (72.0)	503 (79.5)	2310 (80.9)
Malay	45 (11.5)	49 (7.7)	235 (8.2)
Indian	49 (12.5)	62 (9.8)	227 (7.9)
Others	16 (4.1)	19 (3.0)	85 (3.0)
**Highest level of education**			
Secondary and below	97 (24.7)	125 (19.7)	531 (18.6)
Post-secondary	125 (31.8)	215 (34.0)	942 (33.0)
Graduate/postgraduate	171 (43.5)	293 (46.3)	1384 (48.4)
**Type of housing**			
Publicly owned flat with ≤ 3 rooms	64 (16.3)	92 (14.5)	403 (14.1)
Publicly owned flat with 4–5 rooms	304 (77.4)	432 (68.2)	2054 (71.9)
Privately owned property	25 (6.4)	109 (17.2)	400 (14.0)
**Monthly household income (Singapore dollars)^c^**			
≤ 4999	161 (41.0)	231 (36.5)	994 (34.8)
5000–8999	108 (27.5)	181 (28.6)	827 (28.9)
≥ 9000	124 (31.6)	221 (34.9)	1036 (36.3)
**Occupational status**			
Employed or self-employed	291 (74.0)	450 (71.1)	2067 (72.3)
Unemployed	72 (18.3)	105 (16.6)	433 (15.2)
Studying	30 (7.6)	78 (12.3)	357 (12.5)
**Pre-existing medical conditions^d^**			
Yes	72 (18.3)	103 (16.3)	416 (14.6)
No	321 (81.7)	530 (83.7)	2441 (85.4)
**Trusted sources of information^b^**			
Television	263 (66.9)	364 (62.1)	1621 (63.6)
Radio	139 (35.4)	172 (29.4)	816 (32.0)
Print media	211 (53.7)	284 (48.5)	1301 (51.0)
Family and/or relatives	183 (46.6)	300 (51.2)	1242 (48.7)
Friends and/or colleagues	215 (54.7)	351 (59.9)	1496 (58.7)
Social media	288 (73.3)	452 (77.1)	1991 (78.1)
Websites	208 (52.9)	346 (59.0)	1478 (58.0)

COVID-19: coronavirus disease 2019.

^a^ Recruited before the COVID-19 pandemic to a cohort study on infectious disease perceptions.^12^

^b^ More than one response could be selected in “Trusted sources of information”, so percentages do not add up to 100%. Not all questions were asked during the initial survey, so some responses were missing for this; 586 for total cohort and 2550 for total responses.

^c^ One Singapore dollar was corresponding to 0.70 United States dollars in April 2020.

^d^ Including diabetes, hypertension, hyperlipidaemia and asthma.

Regarding sources of information, respondents could select more than one response. A total of 77.1% (452/586) of respondents selected social media as a trusted source of information on infectious disease outbreaks, followed by television programmes (62.1%; 364/586) and friends and/or colleagues (59.9%; 351/586; Table 2).

### Perceptions and knowledge 

**Fig. 2:**
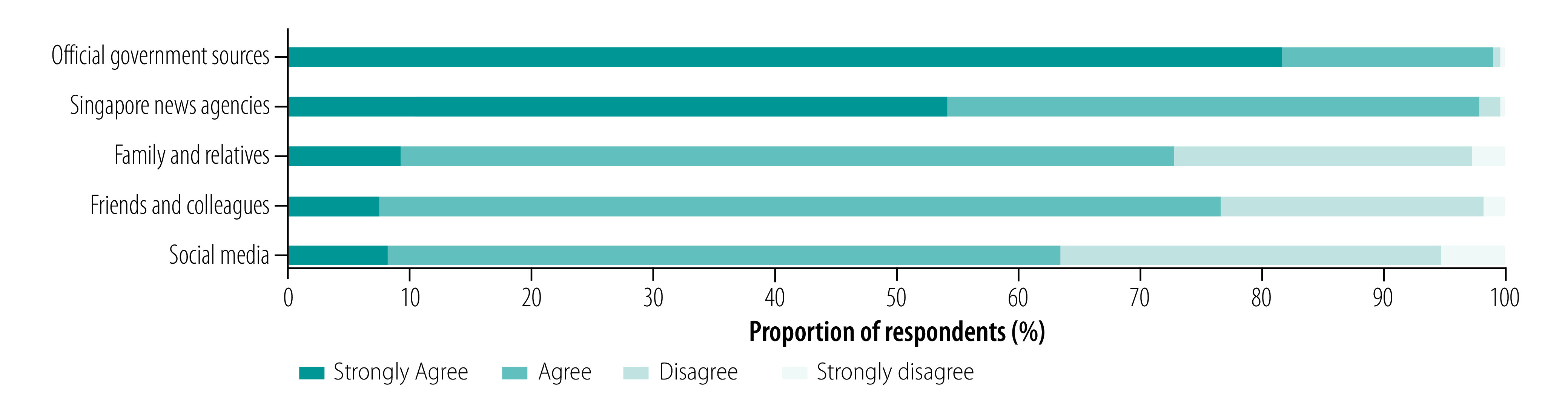
Perceptions of trustworthiness of information sources during COVID-19 pandemic, Singapore, January–April 2020

Most respondents agreed or strongly agreed that information from official government sources (99.1%; 528/533) and Singapore-based news agencies (97.9%; 522/533) was trustworthy (Fig. 2). A sizeable majority also rated information from family and/or relatives (72.8%; 388/533), friends and/or colleagues (76.5%; 408/533) and social media (63.4%; 338/533) as trustworthy. 

COVID-19: coronavirus disease 2019.

**Fig. 3:**
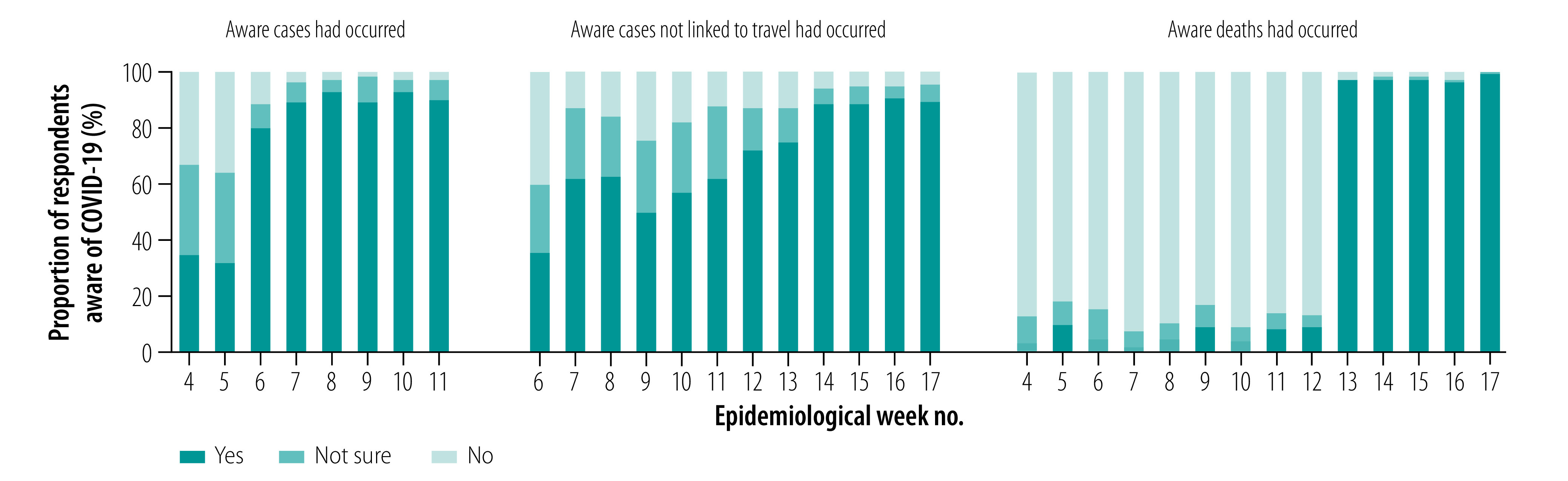
Changes in COVID-19 knowledge among survey respondents, Singapore, January–April 2020

**Fig. 4:**
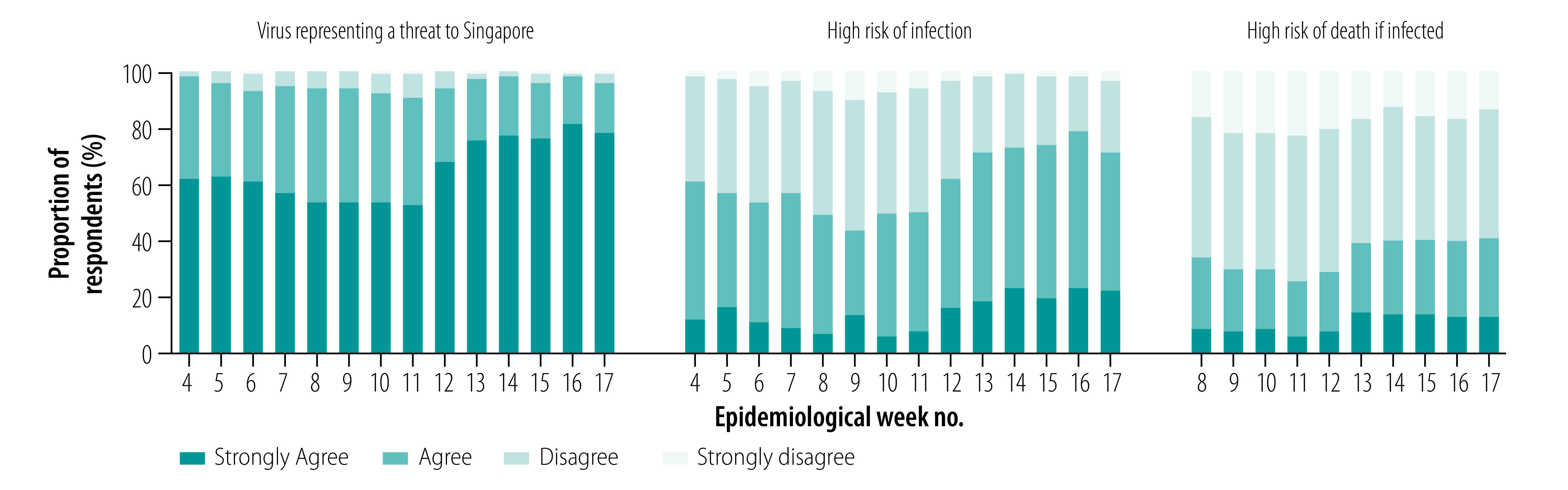
Changes in COVID-19 risk perceptions among survey respondents, Singapore, January–April 2020

Overall, participants demonstrated a high level of knowledge of COVID-19 and its associated symptoms (data repository).^13^ Awareness of the local COVID-19 situation was highly dynamic and reflected current events (Fig. 3). In the first COVID-19 survey, about one third answered that transmission had occurred in Singapore, but this increased to 79.8% (217/272) in week 6 after the first local cluster was reported. Similarly, following the first two fatalities on the last day of week 12, 97.4% (114/117) of the cohort participants were aware of this the following week. Almost all respondents (90.9%; 170/187 to 98.2%; 214/218) agreed the virus is a threat to Singapore; 43.5% (57/131) to 78.9% (172/218) and 25.0% (40/160) to 40.8% (126/309) agreed there was a high chance of becoming infected and, if infected, a high chance of dying, respectively (Fig. 4). These proportions fluctuated with a slight dip during weeks 8–11, before increasing substantially over weeks 12–17 with trends that tracked changes in incident cases and cumulative deaths.

COVID-19: coronavirus disease 2019.

COVID-19: coronavirus disease 2019.

### Trust and behaviour

**Fig. 5:**
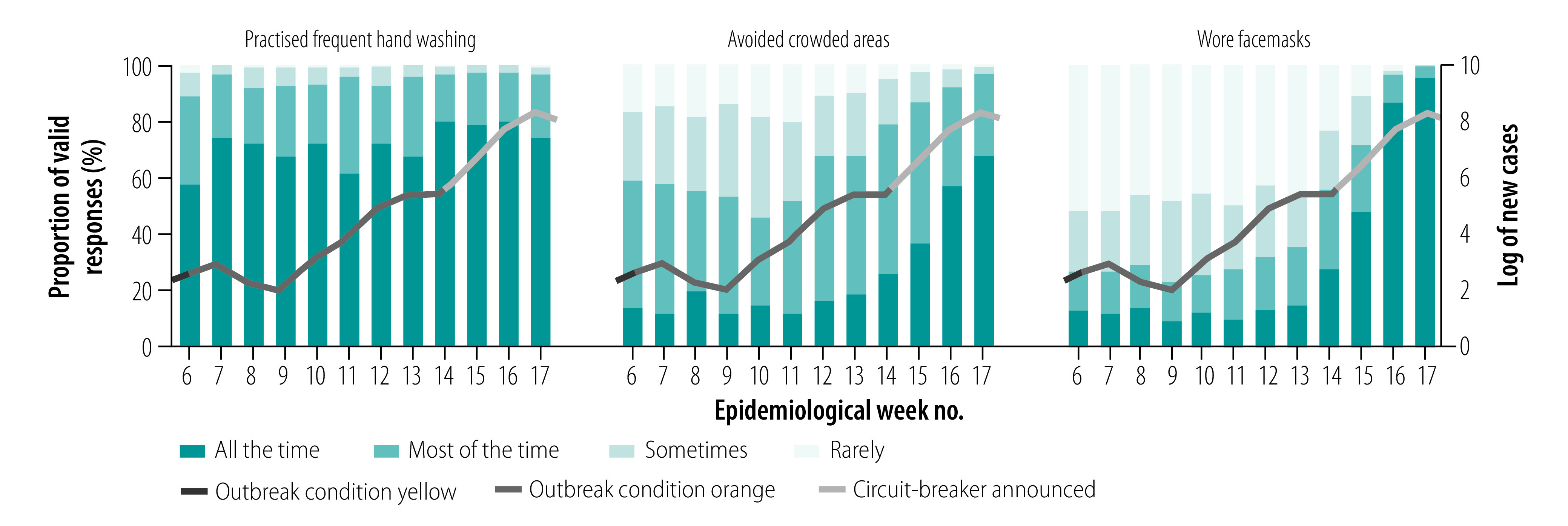
Protective behavioural changes among survey respondents during the COVID-19 pandemic in Singapore, January–April 2020

Most respondents (93.5%; 202/216 to 98.9%; 185/187) agreed or strongly agreed that they could trust the government to communicate the facts about COVID-19 (data repository).^13^ Regarding pandemic-related changes in behaviour, the likelihood of being socially responsible when infected was consistently high from weeks 4 to 13 (data repository).^13^ Almost all (88.6%; 186/210 to 97.4%; 301/309) reported regular hand washing most or all of the time, but the proportions who avoided crowded places and wore facemasks increased substantially during weeks 10–13 from 45.5% (92/202) to 96.8% (299/309) and from 35.8% (43/120) to 99.0% (306/309), respectively (Fig. 5). An increasing proportion reported changing their plans, and 90.5% (57/63) of those who had planned to travel out of Singapore had changed their plans by week 13 (data repository).^13^

COVID-19: coronavirus disease 2019.

Note: Superimposed curve indicates the log of new cases and the different colours represent the disease outbreak response system condition.^14^

Regarding views on government-implemented measures, 80.6% (382/474) to 95.5% (512/536) agreed in surveys 2–7 that various government-implemented measures were needed (data repository).^13^ We observed the lowest support for penalties for not complying with social distancing (80.6%; 382/474), and the need for 5 days of sick leave for acute respiratory infections (82.4%; 291/353). Sizeable proportions felt that some measures were either implemented too late or that more should be done.

### Risk-associated factors

**Fig. 6:**
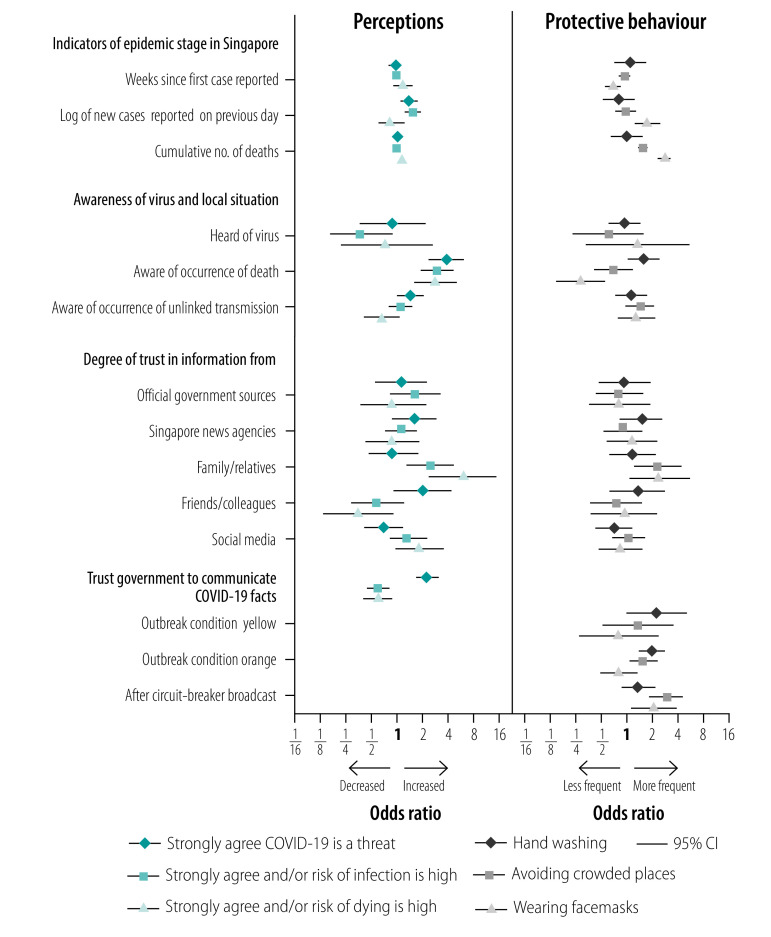
Factors associated with perceptions of risk (strongly agree or agree) and increased frequency of adoption of protective behaviour during COVID-19 pandemic, Singapore, January–April 2020

We evaluated factors associated with the three perception variables of respondents by dichotomizing these responses appropriately (Fig. 6; data repository).^13^

CI: confidence interval.

Note: Multivariable analysis adjusted for recruitment method, age, sex, race, level of education, occupational status, other medical conditions, indicators of epidemic stage in Singapore, awareness of virus and local situation, trust in information from different sources and trust in government to communicate facts during various outbreak phases. Analysis was based on 2517 responses from 572 participants in all cases except for the risk of dying, which was based on 2124 responses from 565 participants.

Participants who enrolled by self-referral were more likely to perceive COVID-19 as a threat compared with those who were recruited during door-to-door interviews. Women were less likely than men to perceive COVID-19 as a threat. Older age and higher educational levels were inversely associated with perceived risk of COVID-19 infection and risk of death if infected, respectively. Multiple indicators of epidemic stage were positively associated with perceptions of threat and risk in univariable analyses (data repository),^13^ but in multivariable analyses the only significant associations were for the log of new cases with increased perceived threat and risk of infection. However, being aware that COVID-19 deaths had occurred was positively associated with perceived threat, risk of infection and risk of death if infected. We observed significant associations between trust in information from family and/or relatives with an increased perceived risk of infection and risk of death if infected. Conversely, trust in information from friends and/or colleagues was significantly associated with a decreased perceived risk of death. In multivariable analyses, trust in government communication on COVID-19 was positively associated with perceived threat (OR: 2.2; 95% CI: 1.6–3.0), but inversely associated with perceived risk of infection (OR: 0.6; 95% CI: 0.4–0.8) and risk of death if infected (OR: 0.6; 95% CI: 0.4–0.9).

All three perception variables were significantly correlated (data repository)^13^ and also significantly associated (*P* < 0.001) with key protective behaviour (data repository)^13^ Given their potential role on causal pathways, these were omitted in multivariable analyses on the frequency of hand washing, avoiding crowded places and wearing facemasks (Fig. 6).

In univariable analysis, significant positive associations between wearing facemasks and both time since the first case and being aware that COVID-19 deaths had occurred (data repository)^13^ became significant inverse associations after adjusting for the log of numbers of new cases and cumulative deaths reported. Our data show that wearing facemasks was positively associated with both the log of number of new cases (OR: 1.8; 95% CI: 1.2–2.5) and the log of number of cumulative deaths reported (OR: 2.9; 95% CI: 2.4–3.5); avoiding crowded places was also positively associated with the log of number of cumulative deaths reported (OR: 1.6; 95% CI: 1.4–1.8). Trust in information from family and/or relatives was positively associated with avoiding crowded places and wearing facemasks. Trust in government communication on COVID-19 was positively associated with hand washing both before and after the declaration of outbreak condition orange; we only observed significant positive associations with avoiding crowded places after the declaration of outbreak condition orange and the circuit-breaker broadcast. Regarding the wearing of facemasks, an initially nonsignificant inverse association (OR < 1.0) became a significant positive association (OR: 2.1; 95% CI: 1.2–3.9) after the circuit-breaker broadcast (data repository).^13^

Deeper questioning about facemasks in surveys 3 and 7 revealed that those with greater trust in government communication were more likely to agree with both the earlier recommendation that facemasks were mainly for those who were sick, and the revised recommendation for facemasks to be worn in public at all times (data repository),^13^ with the association being statistically significant (OR: 2.9; 95% CI: 1.8–4.8; data repository).^13^ We also observed significant positive associations between trust in government communication on COVID-19 and other socially responsible behaviour, such as the covering of mouths when coughing and avoiding social gatherings.

## Discussion

Our online survey approach allowed the public’s knowledge of, and support for, the recommendations and actions of health authorities to be rapidly assessed, and allowed us to examine the factors influencing behaviour adopted in response. We have demonstrated how the perceptions and behaviour of individuals are strongly influenced by the combination of local outbreak conditions and trust in the authorities’ communication on COVID-19.

Overall, respondents demonstrated high levels of knowledge of the current COVID-19 outbreak, which improved further as the pandemic progressed. We have therefore shown that real-time feedback via repeated surveys can help to identify aspects needing clarification or more emphasis in public messaging campaigns. We found that most respondents were up to date with local developments (e.g. the surge in proportions of respondents being aware that deaths had been reported). Variables reflecting counts of cases and deaths and awareness of local deaths were important predictors of perceived threat and risk, as well as the likelihood of adopting key protective behaviour such as avoiding crowded places and wearing facemasks. This result emphasizes the role of timely and accurate detection of infections, as well as transparent reporting of local cases and deaths, in ensuring compliance with public health recommendations. Since the first COVID-19 case in Singapore, health authorities have been using social messaging platforms (e.g. WhatsApp, Facebook) to communicate with the public about the outbreak on a daily basis and to provide advice on how to reduce the risk of infection.^19^ Provision of daily outbreak-related messages from the government to the public could explain why those with greater trust in government communication recognized the threat from COVID-19, and yet perceived their risk of infection and death to be lower, whereas greater trust in information from family and/or relatives appeared to lead to increased perceptions of risk.

Our other noteworthy finding was how trust in government communication on COVID-19 influenced the risk-avoiding behaviour of the public. While trust in advice from family and/or relatives influenced behaviour, greater trust in government communication had temporally nuanced associations corresponding to when this behaviour was recommended by health authorities. In particular, earlier messaging had largely convinced the public that facemasks were only required for those who were sick (data repository).^13^ Subsequently, increased local transmission, and emerging evidence on the role of pre-symptomatic infection^20^ and how facemasks can reduce the transmission of the virus,^21^ led to a decision to recommend universal facemask use in public places. This messaging was largely successful in that it correlated with a substantial rise in facemask use from week 14, even before penalties for non-compliance were introduced in the latter half of week 15. Changes to public information may be needed in an evolving pandemic where new evidence emerges and risk assessments change. We note that, unlike advice from family and/or relatives, government messaging must be rationally calibrated. For instance, when there is evidence that transmission is increasing, health authorities may need to proactively escalate measures even if the public perceives the risk of infection to be low. Health authorities may also need to de-escalate more disruptive measures that are no longer warranted, even when public fears persist. Maintaining public trust in health authorities, in both their response and communications, is therefore crucial during an outbreak so that protective behaviour that is appropriate to the situation is adopted.^22^

Given the complexities of communicating the need for outbreak interventions, a cohort-based approach such as ours has several advantages. Unlike post-outbreak studies,^23^^,^^24^ we were able to influence real-time decision-making and facilitate improvements in communication strategies, allowing authorities to influence public acceptance. While cross-sectional studies during the COVID-19 outbreak have yielded insights on public adoption of preventive measures,^25^^,^^26^ a cohort-based study can track changes in the perceptions and opinions of individuals and also disentangle the underlying effects of participant characteristics and awareness of outbreak developments.

However, the representativeness of our cohort was an important limitation. Reliance on self-referrals and online survey responses, as opposed to more traditional methods such as door-to-door recruitment, may have introduced biases, with evidence that those enrolled through self-referrals were more likely to view COVID-19 as a threat. Moreover, the migrant workers most affected in Singapore’s outbreak were not included in our study; such initiatives must also sample neglected but vulnerable populations. Finally, social desirability bias may partially explain some associations between recommended behaviour and trust in government response.

In conclusion, our findings show that trust is a vital commodity when managing an evolving outbreak. While certain behaviour may also be affected by public awareness of mounting infections and deaths, governments should preferably launch evidence-based public messaging campaigns and interventions ahead of such adverse outcomes. Such information campaigns may be especially required when they run counter to prevailing public opinions, and well-executed behavioural cohort studies can contribute by anticipating the interplay between perceptions, trust and behaviour.
